# Association of lipid profiles and the ratios with arterial stiffness in middle-aged and elderly Chinese

**DOI:** 10.1186/1476-511X-13-37

**Published:** 2014-02-20

**Authors:** Weiwei Zhao, Wei Gong, Nan Wu, Yintao Li, Kuanping Ye, Bin Lu, Zhaoyun Zhang, Shen Qu, Yiming Li, Yehong Yang, Renming Hu

**Affiliations:** 1 Department of Endocrinology and Metabolism, Huashan Hospital, Fudan University, Shanghai, China; 2 Department of Endocrinology, Shanghai Tenth People’s Hospital, School of medicine, Tongji University, Shanghai, China

**Keywords:** Lipid profiles, Arterial stiffness, Brachial-ankle pulse wave velocity (baPWV)

## Abstract

**Background:**

Serum lipids and the ratios are known to be associated with the cardiovascular diseases (CVD). However, the associations of serum lipids and the ratios related to arterial stiffness are unclear. We sought to compare the strength of these serum lipids and the ratios with arterial stiffness assessing by brachial-ankle pulse wave velocity (baPWV) in the middle-aged and elderly Chinese subjects.

**Methods:**

A total number of 1133 Chinese aged from 50 to 90 years old were recruited from Shanghai downtown district. The serum lipids, baPWV and major cardiovascular risk factors of the participants were measured.

**Results:**

Participants with high baPWV exhibited higher levels of non-HDL-c, TC/HDL-c, TG/HDL-c, LDL-c/HDL-c, and non-HDL-c/HDL-c, while HDL-c worked in the opposite direction (all P<0.05). In addition, TC, TG, LDL-c, non-HDL-c, TC/HDL-c, TG/HDL-c, LDL-c/HDL-c, and non-HDL-c/HDL-c had a positive relationship with the baPWV value, while HDL-c was on the contrary (all P <0.05). Finally, individuals with high non-HDL-c/HDL-c (OR 1.71, 95% CI 1.06-2.55, P = 0.013) and low HDL-c (OR 0.57, 95% CI 0.35-0.96, P = 0.024) were seem to be at high risk of arterial stiffness.

**Conclusions:**

As a risk indicator, non-HDL-c/HDL-c, which could be readily obtained from routine serum lipids, was significantly associated with baPWV. Non-HDL-c/HDL-c was superior to traditional lipid variables for estimating arterial stiffness in the middle-aged and elderly Chinese population.

## Introduction

As cardiovascular diseases (CVD) have been the leading cause of death worldwide, the identification of cardiovascular risk factors at an early stage of life has become more and more popular. Some fixed factors (age, sex, and family history) and a number of modifiable factors (hyperlipidemia, smoking, hypertension, hyperglycemia, etc.) had been emerging for predicting CVD [1].

Dyslipidemia is characterized with elevated total cholesterol (TC), triglyceride (TG), and low-density lipoprotein cholesterol (LDL-c) as well as decreased high-density lipoprotein cholesterol (HDL-c) concentrations. The serum lipid abnormalities are recognized as independent approximate risks for CVD. Many investigations had demonstrated the relationships between TC, TG, HDL-c, LDL-c and CVD [2-5]. Arterial stiffness measured by brachial-ankle pulse wave velocity (baPWV) is known to be a marker of subclinical atherosclerosis and serves as an independent risk predictor for CVD [6]. The identification of arterial stiffness in these high risk individuals before the development of clinical atherosclerosis would be of substantial clinical benefit.

We had identified serum HDL-c that had protective effect on arterial stiffness in middle aged and elderly Chinese [7]. Several other cross-sectional studies had also evaluated the strength of the relationships between arterial stiffness and single proatherogenic lipid or ratio, such as the LDL-c, non-HDL-c, and TG/HDL-c. However, the superiority of the lipid parameters was not confirmed and was questioned by different studies [8-10]. In one Chinese study, LDL-c was found independently associated with aortic stiffness [8]. Furthermore, a Netherlandish study supported the use of non-HDL-c as a superior predictor to LDL-c in identifying individuals for arterial stiffness [9]. An American study showed that TG/HDL-c was an independent determinant of arterial stiffness in adolescents and young adults [10]. On these premises, it prompted us to perform the present study to directly compare the association of these serum lipids to the ratios (including TC, TG, HDL-c, LDL-c, non-HDL-c, TC/HDL-c, TG/HDL-c, LDL-c/HDL-c, and non-HDL-c/HDL-c) with arterial stiffness assessing by baPWV in the middle-aged and elderly Chinese population in an attempt to optimize the risk factors.

## Methods

### Study subjects

From September 2007 to August 2008, a large-scale local health-check program was launched by Huashan Hospital in Shanghai, China. All studied individuals came from the Jiangninglu community of the Jingan District and the Youyilu community of the Baoshan District in Shanghai. A multistage stratified cluster sampling method was carried out to select subjects from the two communities. We selected the units in neighborhood. Only participants aged over 50 years old were recruited from each household. All studied individuals were of southern Han Chinese. A total of 1492 eligible subjects participated in the study, 359 of them were excluded from the analysis for the usage of hypolipidemic agents or missing data. Written informed consents were obtained from all participants and the study was approved by the Institutional Review Board of Huashan Hospital.

Participants with the following conditions were excluded: nutritional derangements; anaemia; malignancy; thyroid dysfunction; pregnancy; breast-feeding; mental illness.

### Data collection

The methodology used to obtain data of clinical and biochemical variables had been previously published [7]. In brief, a standardized questionnaire was performed by qualified internist to collect information such as age, gender, smoking, self-reported dyslipidemia, hypertension, and CVD.

All subjects were assessed after overnight fasting for at least 10 hours. The details of anthropometric measurements include height, weight, waist circumference, hip circumference, and blood pressure were carried by trained physicians using standard methods. Body mass index (BMI) was calculated as weight in kilograms divided by the square of height in meters.

### Laboratory measurements

The measurements of fasting plasma glucose and hemoglobin A1c, fasting serum insulin, TC, TG, HDL-c, LDL-c, creatinine, uric acid, and white blood cell counts were performed in the centralized laboratory of Huashan Hospital [7,11]. Non-HDL-c was calculated by subtracting HDL-c from TC. Insulin resistance was estimated by homeostasis model assessment index-insulin resistance (HOMA-IR) [12]. Glomerular filtration rate (GFR; expressed in ml/min/1.73 m^2^) was calculated by CKD-EPI equation [13].

Hypertension was defined as if blood pressure 140/90 mmHg or greater or current use of antihypertensive drugs [14]. Metabolic syndrome (Mets) was defined based on the updated International Diabetes Federation (IDF) criteria for Asian-American [15].

### Measurements of baPWV

BaPWV was measured by the VP-1000 Automatic Arteriosclerosis Measurement System (model BP-203 RPE-II, Colin Co, Ltd, Komaki, Japan). The details of the measurement, validity, and reproducibility have been reported previously [16,17]. The correlation coefficients of interobserver and intraobserver reproducibility were 0.98 and 0.87, respectively. The device can record pulse waves in four cuffs, record the transmission time to arm and ankles, calculate the transmission distance from the right arm to each ankle according to body height, and automatically compute the baPWV values by transmission time and distance. Measurements were taken after the subject had a 5 min rest in a supine position at room temperature. Participants were advised to refrain from smoking, alcohol, caffeinated beverages and vigorous exercise for over 2 hours prior to the examination. The relationship between left and right baPWV was pretty high (r = 0.955, P < 0.001) [7]. As a result, we used the mean of bilateral baPWV value during analysis.

### Statistical analysis

All statistical analysis was performed using SPSS version 16.0 (SPSS, Chicago, IL, USA). Continuous variables were expressed as means ± SD or median (interquartile range). Categorical variables were represented by frequency and percentage. The characteristics of the study population and lipid parameters according to baPWV tertiles were compared by ANOVA for continuous variables and chi-squared test for categorical variables. Correlation coefficients between lipid parameters and baPWV were calculated by partial correlation analysis. The odds ratios for high baPWV (defined as baPWV >75th percentile) [7,18] were calculated by a multivariate logistic regression analysis in different lipid parameters. We stratified the lipids and the ratios into tertiles and estimated the odds ratios for developing arterial stiffness among different tertiles by using the first tertile as reference.

## Results

### General characteristics of the study participants

1133 participants were analyzed, among which 38.0% (430/1133) were men, 62.0% (703/1133) were women. Table 1 compared the demographic, anthropometric, and biochemical characteristics of the experimental population, which was divided into different gender subgroups. The levels of age, waist to hip ratio, diastolic blood pressure, TC/HDL-c, LDL-c/HDL-c, nonHDL-c/HDL-c, HbA1C, white blood cell, serum creatinine, uric acid, and the percentage of smoking were significantly higher in men than in women. The levels of TC, HDL-c, LDL-c, nonHDL-c, GFR, baPWV, and the state of MetS were significantly lower in men than in women (all P <0.05). The level of body mass index, systolic blood pressure, TG, TG/HDL-c, and the percentage of self-reported CVD, the state of hypertension were similar in both genders.

**Table 1:** Characteristics of participants categorized by gender classification

	**Total**	**Male**	**Female**	**P**
Number	1133	430	703	
Age (years)	64.0 ± 8.6	65.4 ± 8.7	63.1 ± 8.4	<0.001
Smoking (%)	246(21.7)	136(31.6)	110(15.6)	<0.001
Self-reported CVD (%)	239(21.1)	83(19.3)	156(22.2)	0.279
Waist to hip ratio	0.88 ± 0.06	0.91 ± 0.06	0.86 ± 0.06	<0.001
Body mass index (kg/m^2^)	25.4 ± 3.3	25.2 ± 3.2	25.5 ± 3.3	0.278
SBP (mmHg)	138.7 ± 19.2	139.0 ± 18.8	138.5 ± 19.4	0.630
DBP (mmHg)	83.8 ± 10.6	85.0 ± 10.1	83.0 ± 10.8	0.002
Hypertension (%)	809(71.4)	317(73.7)	492(70.0)	0.200
TC	5.34 ± 1.00	5.08 ± 0.91	5.50 ± 1.03	<0.001
TG	1.61(1.09-2.30)	1.55(1.06-2.20)	1.65(1.10-2.38)	0.154
HDL	1.29 ± 0.32	1.21 ± 0.31	1.34 ± 0.32	<0.001
LDL	3.02 ± 0.78	2.91 ± 0.73	3.09 ± 0.80	0.001
nonHDL	4.04 ± 0.97	3.87 ± 0.91	4.15 ± 0.99	<0.001
TC/HDL	4.32 ± 1.11	4.41 ± 1.14	4.27 ± 1.09	0.043
TG/HDL	1.29(0.80-2.05)	1.36(0.86-2.11)	1.27(0.77-1.98)	0.613
LDL/HDL	2.44 ± 0.79	2.53 ± 0.84	2.39 ± 076	0.005
nonHDL/HDL	3.32 ± 1.11	3.41 ± 1.14	3.27 ± 1.09	0.044
FPG (mmol/l)	6.9 ± 2.3	6.9 ± 2.5	6.8 ± 2.1	0.508
HbA1C	6.6 ± 1.3	6.8 ± 1.5	6.5 ± 1.2	0.021
HOMA-IR	3.37(2.12-5.56)	3.33(2.13-5.92)	340(2.10-5.44)	0.637
GFR (ml/min/1.73^2^)	92.2 ± 13.7	89.5 ± 14.5	93.9 ± 13.0	<0.001
BaPWV (cm/s)	1745 ± 364	1782 ± 364	1723 ± 362	0.040
MetS (%)	759(67.0)	247(57.4)	512(72.8)	<0.001
White blood cell (10^9^/l)	6.1 ± 1.6	6.4 ± 1.7	5.9 ± 1.4	<0.001
Serum creatinine (μmol/l)	64.3 ± 18.7	76.4 ± 21.7	56.9 ± 11.4	<0.001
Uric acid (μmol/l)	315(260–370)	345(290–400)	300(251–347)	0.007

CVD, cardiovascular disease; SBP, systolic blood pressure; DBP, diastolic blood pressure; FPG, fasting plasma glucose; HOMA-IR, homeostasis model assessment index-insulin resistance; GFR, glomerlar filtration rate; BaPWV, brachial-ankle pulse wave velocity; MetS, metabolic syndrome.

Data were reported in mean ± SD, median (interquartile range), or number (percent).

### Lipid profiles distribution according to baPWV tertiles

The participants were divided into tertiles on the basis of the levels of baPWV. The lipid profiles and the ratios distribution of the participants differing by baPWV tertiles were performed in Table 2. In the overall and female population, participants with high baPWV exhibited higher levels of non-HDL-c, TC/HDL-c, TG/HDL-c, LDL-c/HDL-c, and non-HDL-c/HDL-c, while HDL-c worked in the opposite direction (P<0.05). However, in men, only the levels of TC/HDL-c and non-HDL-c/HDL-c increased with the level of baPWV, and HDL-c decreased with the level of baPWV (P<0.05).

**Table 2:** Serum lipids and their ratios distribution according to baPWV tertiles

		**T1**	**T2**	**T3**	**P**
TC	Overall	5.29 ± 0.91	5.40 ± 0.98	5.34 ± 1.07)	0.478
(mmol/L)	M	5.06 ± 0.79	5.12 ± 0.86	5.05 ± 0.95	0.597
	F	5.41 ± 0.94	5.55 ± 1.01	5.57 ± 1.12	0.307
TG	Overall	1.51(1.01-2.17)	1.69(1.03-2.37)	1.58(1.11-2.47)	0.045
(mmol/L)	M	1.44(0.99-2.04)	1.56(1.01-2.18)	1.54(1.03-2.24)	0.585
	F	1.51(1.01-2.21)	1.77(1.04-2.47)	1.65(1.23-2.66)	0.014
HDL-c	Overall	1.36 ± 0.33	1.29 ± 0.30	1.24 ± 0.29	<0.001
(mmol/L)	M	1.30 ± 0.39	1.26 ± 0.31	1.15 ± 0.24	0.019
	F	1.39 ± 0.30	1.31 ± 0.29	1.30 ± 0.31	0.009
LDL-c	Overall	2.98 ± 0.72	3.06 ± 0.79	2.98 ± 0.76	0.36
(mmol/L)	M	2.84 ± 0.66	2.92 ± 0.73	2.88 ± 0.73	0.648
	F	3.05 ± 0.74	3.14 ± 0.82	3.06 ± 0.77	0.442
nonHDL-c	Overall	3.92 ± 0.89	4.11 ± 0.97	4.11 ± 1.03	0.043
(mmol/L)	M	3.75 ± 0.79	3.86 ± 0.92	3.90 ± 0.94	0.512
	F	4.00 ± 0.93	4.25 ± 0.97	4.27 ± 1.07	0.03
TC/HDL-c	Overall	4.05 ± 0.99	4.35 ± 1.07	4.49 ± 1.15	<0.001
	M	4.14 ± 1.05	4.29 ± 1.18	4.56 ± 1.14	0.032
	F	4.01 ± 0.96	4.39 ± 1.02	4.44 ± 1.17	<0.001
TG/HDL-c	Overall	1.09(0.68-1.89)	1.35(0.77-2.03)	1.37(0.86-2.19)	0.003
	M	1.21(0.74-2.08)	1.33(0.77-1.95)	1.40(0.84-2.24)	0.187
	F	1.07(0.63-1.83)	1.38(0.78-2.08)	1.33(0.86-2.17)	0.005
LDL-c/HDL-c	Overall	2.29 ± 0.74	2.47 ± 0.78	2.50 ± 0.75	0.004
	M	2.35 ± 0.78	2.48 ± 0.90	2.58 ± 0.78	0.158
	F	2.26 ± 0.71	2.47 ± 0.71	2.44 ± 0.72	0.012
nonHDL-c/HDL-c	Overall	3.04 ± 0.98	3.40 ± 1.07	3.50 ± 1.15	<0.001
	M	3.13 ± 1.05	3.28 ± 1.18	3.56 ± 1.14	0.027
	F	3.00 ± 0.95	3.39 ± 1.02	3.45 ± 1.16	<0.001

TC, total cholesterol; TG, triglyceride; HDL-c, high-density lipoprotein cholesterol.

LDL-c, low-density lipoprotein cholesterol; baPWV, brachial-ankle pulse wave velocity; T, tertile; M, male; F, female.

### The relationships between baPWV and lipid profiles

As some lipid parameters were differently distributed in men and women, we investigated the relationships of serum lipids and lipid ratios with baPWV in men and woman separately. As shown in Table 3, baPWV was significantly associated with TC, TG, HDL-c, LDL-c, non-HDL-c, TC/HDL-c, TG/HDL-c, LDL-c/HDL-c, and non-HDL-c/HDL-c in both genders. The highest correlation coefficient reached 0.180 in non-HDL-c/HDL-c. However, the correlationship of TC and TG with baPWV disappeared in male, while the correlationship of TC and LDL-c with baPWV disappeared in female.

**Table 3:** Correlation between baPWV and lipid parameters

**Variable**	**Overall**^**b**^	**Male**^**c**^	**Female**^**c**^
TC	0.074^a^	0.094	0.062
TG	0.124^a^	0.102	0.132^a^
HDL-c	−0.143^a^	−0.140^a^	−0.162^a^
LDL-c	0.075^a^	0.124^a^	0.047
nonHDL-c	0.125^a^	0.171^a^	0.096^a^
TC/HDL-c	0.177^a^	0.231^a^	0.130^a^
TG/HDL-c	0.136^a^	0.133^a^	0.133^a^
LDL-c/HDL-c	0.154^a^	0.217^a^	0.106^a^
Non-HDL-c/HDL-c	0.180^a^	0.240^a^	0.135^a^

TC, total cholesterol; TG, triglyceride; HDL-c, high-density lipoprotein cholesterol.

LDL-c, low-density lipoprotein cholesterol; BaPWV, brachial-ankle pulse wave velocity.

^a^P < 0.05.

^b^Correlation coefficients were calculated after adjusted for age and gender.

^c^Correlation coefficientswere calculated after adjusted for age.

Remarkably, as presented in the multivariate logistic regression analysis, the ORs for probability of being arterial stiffness were increased along non-HDL-c/HDL-c (OR 1.71, 95% CI 1.06-2.55, P = 0.013) tertiles after adjusting for age and sex, and decreased along HDL-c (OR 0.57, 95% CI 0.35-0.96, P = 0.024) tertiles (Table 4). However, the ORs of TC, TG, LDL-c, non-HDL-c, TC/HDL-c, TG/HDL-c, and LDL-c/HDL-c did not reach statistical significance.

**Table 4:** Age and sex-adjusted odds ratios (95% confidence intervals) for high baPWV by tertiles of lipid profiles

**Tertiles**	**Odds Ratio (95% confidence interval)**				
	**Age and sex-adjusted**				
	TC	TG	HDL-c	LDL-c	Non-HDL-c
I	1	1	1	1	1
II	1.12(0.71-1.75)	1.27(0.81-1.99)	0.71(0.46-1.08)	1.12(0.70-1.77)	1.12(0.72-1.74)
III	1.27(0.85-1.92)	1.38(0.91-2.11)	0.57(0.35-0.96)	1.40(0.90-2.16)	1.27(0.84-2.04)
P	0.501	0.185	0.024	0.104	0.394
	TC/HDL-c	TG/HDL-c	LDL-c/HDL-c	Non-HDL-c/HDL-c	
I	1	1	1	1	
II	1.07(0.68-1.69)	1.16(0.74-1.83)	1.44(0.92-2.26)	1.08(0.75-1.67)	
III	1.56(1.00-2.43)	1.52(0.97-2.37)	1.52(0.96-2.40)	1.71(1.06-2.55)	
P	0.050	0.069	0.075	0.013	

TC, total cholesterol; TG, triglyceride; HDL-c, high-density lipoprotein cholesterol.

LDL-c, low-density lipoprotein cholesterol; BaPWV, brachial-ankle pulse wave velocity.

## Discussion

The study was conducted in a population of middle-aged and elderly Chinese, which was known to have a high prevalence of arterial stiffness. In previous study, we concluded that serum HDL-c had protective effect on arterial stiffness. As an extension of previous study, we investigated the clinical evaluation of the various lipid ratio profiles and had two major findings: 1) all the lipid profiles and lipid ratios, such as TC, TG, HDL-c, LDL-c, non-HDL-c, TC/HDL-c, LDL-c/HDL-c, and non-HDL-c/HDL-c, are significantly correlated with arterial stiffness assessing by baPWV, and 2) Individuals with higher non-HDL-c/HDL-c levels have higher risk of arterial stiffness than other lipid parameters.

CVD represents a major cause of morbidity and mortality worldwide [1]. Arterial stiffness is an important determinant of cardiovascular risk [19,20]. Patients with hypercholesterolaemia have higher stiff blood vessels than matched controls, which have similar peripheral blood pressures [21]. Earlier studies had indicated that lipids and the related ratios were strong predictors of CVD. Some lipids had been the main target for clinical intervention of CVD [22-25]. Most previous studies focused on only one ratio or traditional lipids [8-10]. There is no study that evaluated the relationship between all the lipids and lipid ratios with arterial stiffness. In the present study, we evaluated the relationship between the lipids and the ratios with baPWV, and highlighted the rationale for using the lipid ratio of non-HDL-c/HDL-c as a risk factor for arterial stiffness risk in middle-aged and elderly Chinese.

In the study, HDL-c and all the lipid ratios were significantly correlated with the baPWV tertiles in the overall patients. Only HDL-c, TC/HDL-c, and non-HDL-c/HDL-c worked in both the male and female population. All the conventional lipids and the ratios had a significant correlation with baPWV in the overall population. Only HDL-c and the lipid ratios worked in both the male and female population. Finally, in the multivariate logistic regression analysis after adjusting for age and gender, only non-HDL-c/HDL-c and HDL-c had significant estimating capacities. Non-HDL-c/LDL-c seemed to be superior to HDL-c in assessing the risk for arterial stiffness. Non-HDL-c/HDL-c, an easily obtained index, maybe offer convenience in screening large populations of arterial stiffness risk in routine clinical practice.

The pathophysiology of non-HDL-c/HDL-c with arterial stiffness is unclear, but data from literatures is helpful. Non-HDL-c is a measurement of the cholesterol in LDL and VLDL (intermediate-density lipoprotein and very-LDL) particles. In clinical practice, non-HDL-c had been recommended as a secondary therapeutic target in individuals with high TG concentration. It also had been reported to be a better predictor of CVD than LDL-c [26]. The non-HDL-c/HDL-c is a lineal combination of TG and HDL-c. In the last few years, the focus had been shifted towards non-HDL-c/HDL-c. A report from the Swedish National Diabetes Register demonstrated that non-HDL-c/HDL-c had a stronger relationship with coronary heart disease risk than LDL-c, HDL-c and non-HDL-c. A recent study found that non-HDL-c/HDL-c was a better indicator than apolipoprotein B (ApoB)/apolipoprotein A1 (ApoA1) and ApoB/LDL-c in identifying MetS and insulin resistance [27,28]. However, few studies had evaluated the relationship between non-HDL-c/HDL-c ratio and arterial stiffness.

The study compared the association between the routine lipids and the related ratios with baPWV for the first time. However, the study has some limitations. First, the experimental population was modest in size, and entirely of Chinese origin, which may be limited in the generalizability of the results. Second, since the study was cross-sectional designed, no causal relationship between lipid profiles and the risk of arterial stiffness can be drawn. Thus, we hope the findings can stimulate more investigators.

In conclusion, the study provides the novel findings that all the lipids and the ratios are significantly correlated with baPWV. Non-HDL-c/HDL-c ratio performs more effectively in identifying individuals at increased arterial stiffness risk. As non-HDL-c/HDL-c is easy and cost-effective obtained, it seems reasonable to propose non-HDL-c/HDL-c as a surrogate indicator of arterial stiffness in clinical practice.

## Abbreviations

baPWV: Brachial-ankle pulse wave velocity; TC: Total cholesterol; TG: Triglyceride; LDL-c: Low-density lipoprotein cholesterol; HDL-c: High density lipoprotein-cholesterol; CVD: Cardiovascular diseases; BMI: Body mass index; HOMA-IR: Homeostasis model assessment index-insulin resistance; GFR: Glomerlar filtration rate; MetS: Metabolic syndrome; VLDL: Intermediate-density lipoprotein and very-LDL; ApoB: Apolipoprotein B; ApoA1: Apolipoprotein A1.

## Competing interests

The authors declare that they have no competing interests.

## Authors’ contributions

YY and RH designed the study; WZ, NW, YL and KY participated in acquisition of data; BL, ZZ, SQ and YL researched and evaluated the literature; WZ and WG undertook the statistical analysis and wrote the first draft of the manuscript. All authors have approved the final manuscript for publication.
